# Safety and Pharmacokinetics of the Aminomethylcycline Antibiotic Omadacycline Administered to Healthy Subjects in Oral Multiple-Dose Regimens

**DOI:** 10.1128/AAC.01487-17

**Published:** 2018-01-25

**Authors:** Lu Ann Bundrant, Evan Tzanis, Lynne Garrity-Ryan, Stephen Bai, Surya Chitra, Amy Manley, Stephen Villano

**Affiliations:** a PPD Phase I Clinic, Austin, Texas, USA; b Paratek Pharmaceuticals, Inc., King of Prussia, Pennsylvania, USA

**Keywords:** multidose regimen, omadacycline, oral dosing

## Abstract

Omadacycline, a first-in-class aminomethylcycline antibiotic, is related to tetracyclines but is structurally modified to circumvent mechanisms of resistance to tetracyclines. Omadacycline demonstrates potent activity against a broad range of pathogens, including drug-resistant strains, and is in late-stage development for treatment of acute bacterial skin and skin structure infections and community-acquired bacterial pneumonia. Previous studies support an intravenous-to-oral transition regimen with 300-mg once-daily oral dosing. This phase 1 study investigated the pharmacokinetics and safety/tolerability of multiple oral omadacycline doses higher than 300 mg. Using a 3-period crossover design, healthy adults were randomized to receive oral omadacycline at 300, 450, and 600 mg in variable sequence (*n* = 26) or placebo (*n* = 7) once daily for 5 consecutive days per period. In plasma, omadacycline maximum concentration and total exposure increased with increasing dose but were less than dose proportional. The kinetics of omadacycline plasma accumulation were similar between dose levels; exposure on day 5 was ∼50% higher than that on day 1. Omadacycline plasma concentrations on day 1 of 450-mg dosing were similar to those on day 5 of 300-mg dosing. All doses were generally well tolerated, but the 600-mg dose was associated with more gastrointestinal adverse events.

## INTRODUCTION

Omadacycline is a potent semisynthetic antibiotic that represents a new class of tetracycline-related compounds, the aminomethylcyclines (reviewed in references 1 and 2). Like that of tetracyclines, the antibacterial activity of omadacycline stems from its ability to bind to the tetracycline binding site on the 30S subunit of the bacterial ribosome and inhibit bacterial protein synthesis (3, 4). Notably, however, structural modifications at the C-7 and C-9 positions allow omadacycline to overcome the 2 main mechanisms of resistance to tetracyclines, efflux pumps and ribosomal protection (2, 4, 5). These modifications result in an improved spectrum of activity compared with those of earlier-generation tetracyclines. Omadacycline demonstrates potent *in vitro* activity against Gram-positive and Gram-negative aerobes, anaerobes, and atypical pathogens, which include Legionella and Chlamydia spp. Omadacycline also displays *in vitro* activity against drug-resistant pathogens common in community-acquired infections, such as methicillin-resistant Staphylococcus aureus (MRSA), multidrug-resistant Streptococcus pneumoniae, vancomycin-resistant Enterococcus spp. (VRE), and extended-spectrum beta-lactamase (ESBL)-producing Escherichia coli (2, 6).

Omadacycline is currently in late-stage development as a monotherapy for treatment of acute bacterial skin and skin structure infections (ABSSSI) and community-acquired bacterial pneumonia (CABP), both of which may involve pathogens resistant to current standard-of-care drugs. Both intravenous (i.v.) and oral formulations of omadacycline are in development and have been evaluated in more than 20 phase 1 trials. These phase 1 trials established pharmacokinetic (PK) profiles, proved general safety and tolerability at therapeutic doses, and demonstrated that a 300-mg oral dose provides exposure comparable to that of a 100-mg i.v. dose (2, 7). Phase 3 trials evaluating i.v. to once-daily oral dosing have been completed for both ABSSSI (ClinicalTrials registration no. NCT02378480) and CABP (ClinicalTrials registration no. NCT02531438).

In completed clinical studies, single i.v. and single oral doses up to 600 mg have been investigated (1, 2). Multiple i.v. doses of 100 mg or 200 mg once daily for up to 14 and 7 consecutive days, respectively, and multiple oral doses of 200 mg or 300 mg once daily for up to 10 consecutive days have also been evaluated (1, 2). Based on the results of these studies, the once-daily oral dose selected for use in the phase 3 ABSSSI and CABP trials was 300 mg (following at least 3 days of i.v. omadacycline administration). In both of these trials, i.v. to once-daily oral dosing with omadacycline met primary endpoints and was not inferior to i.v. to oral dosing with comparator antibiotics, with a favorable overall safety and tolerability profile (8, 9). It is possible that a higher oral dose could be used as a loading dose to eliminate the need for an initial i.v. infusion phase of treatment for ABSSSI and CABP. A higher oral dose might also be important for additional potential indications involving bacterial infections in organs/tissues other than skin or lungs. Therefore, this phase 1 study was designed to compare the pharmacokinetics and safety of multiple oral doses of 300, 450, or 600 mg omadacycline when administered to healthy adults once daily for 5 consecutive days. Within this dose range, we hypothesized that the oral pharmacokinetics of omadacycline were linear and that multiple doses of omadacycline generally would be safe and well tolerated.

## RESULTS

### Demographics, baseline characteristics, and disposition of study subjects.

Of the 33 subjects enrolled in the study, 26 were assigned to receive omadacycline and 7 were assigned to receive placebo. Demographic and baseline characteristics were generally similar between omadacycline and placebo treatment groups (Table 1) and across all omadacycline treatment sequences (data not shown). The majority of subjects in the study were white (57.6%) and male (81.8%). The overall mean age of subjects was 36.9 years, with a range of 21 to 55 years.

**TABLE 1:** Demographics and baseline characteristics of subjects in the study^*a*^

Parameter	Value(s) of treatment with:		
	Omadacycline (*n* = 26)	Placebo (*n* = 7)	Overall (*N* = 33)
Age, yr			
Mean (±SD)	35.6 (±10.4)	41.9 (±11.6)	36.9 (±10.8)
Min, max	21, 55	25, 53	21, 55
Sex, *n* (%)			
Male	21 (80.8)	6 (85.7)	27 (81.8)
Female	5 (19.2)	1 (14.3)	6 (18.2)
Race, *n* (%)			
White	15 (57.7)	4 (57.1)	19 (57.6)
Black or African-American	9 (34.6)	3 (42.9)	12 (36.4)
Asian	2 (7.7)	0	2 (6.1)
Height, cm			
Mean (±SD)	173.12 (±9.17)	172.89 (±4.31)	173.07 (±8.32)
Min, max	155.2, 192.4	165.6, 177.4	155.2, 192.4
Weight, kg			
Mean (±SD)	78.67 (±10.33)	83.77 (±4.80)	79.75 (±9.60)
Min, max	62.7, 101.4	76.7, 90.4	62.7, 101.4
Body mass index, kg/m^2^			
Mean (±SD)	26.25 (±2.72)	28.04 (±1.45)	26.63 (±2.59)
Min, max	19.4, 29.8	25.8, 29.9	19.4, 29.9

a Results for safety population.

All 33 subjects received at least 1 dose of study drug (omadacycline or placebo) and were included in the safety analysis population. Twenty-five of the 26 omadacycline-treated subjects (96.2%) were included in the pharmacokinetic (PK) analysis population (1 subject was excluded from this population due to vomiting after dosing). Five subjects (15.2%) discontinued the study early, including 4 omadacycline-treated subjects (15.4%) and 1 placebo-treated subject (14.3%). Four subjects (12.1%) discontinued due to treatment-emergent adverse events (TEAEs), including 3 omadacycline-treated subjects (11.5%) and 1 placebo-treated subject (14.3%), as detailed below. In addition, 1 omadacycline-treated subject was lost to follow-up. Thus, 22 subjects received all 5 doses of 300, 450, and 600 mg omadacycline, and 6 subjects received all 5 doses of placebo in periods 1, 2, and 3. These subjects were considered to have completed the study.

### Plasma pharmacokinetics.

**FIG 1:**
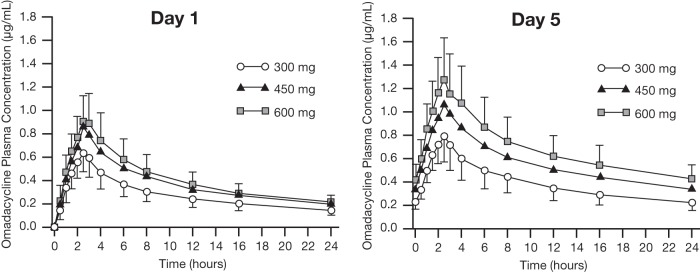
Plasma concentration-versus-time curves of omadacycline after oral administration. Mean (±SD) plasma concentrations of omadacycline versus time are shown by omadacycline dose group (300, 450, or 600 mg) for the PK population. Oral omadacycline doses were administered at time zero on each of 5 consecutive days of dosing in each of 3 periods. Blood samples were collected for PK analysis on day 1 (left) and day 5 (right). Data were pooled by omadacycline dose for all subjects regardless of the period in which they received a particular dose. SD, standard deviations.

At all tested omadacycline dose levels, on both day 1 and day 5 of each 5-day treatment period, mean plasma omadacycline concentrations peaked 2.5 h after dosing (time to reach maximum observed plasma concentration [*T*_max_]), and omadacycline was measurable in plasma for up to 24 h after dosing (the last sampling time). Omadacycline total exposure (area under the plasma concentration-versus-time curve from time 0 to 24 h after dosing [AUC_0–24_] and AUC from time zero to the last quantifiable concentration [AUC_last_]) and peak concentrations (maximum observed plasma concentration [*C*_max_]) increased with increasing omadacycline dose (300 versus 450 versus 600 mg) on both day 1 and day 5 and were higher on day 5 than on day 1 for corresponding doses (Fig. 1 and Table 2). The mean terminal elimination half-life (*t*_1/2_) of omadacycline in plasma was similar across the 3 tested dose levels, ranging from 13.03 to 13.66 h on day 1 and from 15.49 to 16.83 h on day 5. Steady-state conditions appeared to have been reached by day 5, because the concentrations at *t* = 0 and *t* = 24 following the day 5 dose were similar. Between-subject variability in systemic omadacycline exposure was low and was similar at all 3 tested dose levels, with coefficients of variation (CVs) ranging from 23.2% to 26.6% for *C*_max_, AUC_0–24_, and AUC_last_ on day 1 and from 25.0% to 27.1% for *C*_max_, AUC_0–24_, and AUC_last_ on day 5 (Table 2).

**TABLE 2:** Plasma pharmacokinetic parameters of omadacycline, by dose, on days 1 and 5 of dosing^*a*^

Parameter	Value for each omadacycline dose on day:					
	1			5		
	300 mg (*n* = 25)	450 mg (*n* = 24)	600 mg (*n* = 24)	300 mg (*n* = 23)	450 mg (*n* = 24)	600 mg (*n* = 23)
Mean AUC_0–24_, ng · h/ml (CV)	6,644.8 (25.3)	8,976.5 (26.6)	10,020.5 (25.7)	9,267.2 (26.8)	13,366.7 (26.0)	16,420.3 (27.1)
Mean AUC_last_, ng · h/ml (CV)	6,634.2 (25.3)	8,962.5 (26.6)	10,004.5 (25.7)	9,270.2 (26.8)	13,368.3 (25.9)	16,424.6 (27.1)
Mean *C*_max_, ng/ml (CV)	648.8 (24.0)	874.2 (26.6)	954.5 (23.2)	808.8 (25.9)	1,077.3 (25.0)	1,305.5 (26.6)
Mean *T*_max_, h (min, max)	2.50 (1.50, 3.00)	2.50 (1.50, 3.00)	2.51 (1.00, 3.00)	2.50 (1.00, 3.00)	2.50 (1.50, 4.00)	2.50 (2.00, 4.00)
Mean *t*_1/2_, h (CV)	13.66^*b*^ (12.5)	13.45^*c*^ (12.9)	13.03^*c*^ (11.8)	15.49^*d*^ (10.7)	16.83^*c*^ (8.1)	16.75^*d*^ (6.8)

a Results are for the PK population. One subject during 300-mg omadacycline dosing and 1 subject during 600-mg omadacycline dosing vomited before reaching PK steady state on day 5. These subjects met criteria for exclusion from PK analyses and are not included in the day 5 summary.

b *n* = 24 (*t*_1/2_ was not estimable for 1 subject).

c *n* = 23 (*t*_1/2_ was not estimable for 1 subject).

d *n* = 21 (*t*_1/2_ was not estimable for 2 subjects).

Although omadacycline AUC_0–24_, AUC_last_, and *C*_max_ increased with increasing omadacycline dose, the observed increases in exposure were less than dose proportional on both days of analysis (Tables 3 and 4). Statistical analyses showed that with an increase in dose from 300 mg to 600 mg, omadacycline exposure (based on dose-normalized AUC_0–24_) on day 1 was 76% of that predicted if exposure were perfectly dose proportional; on day 5, the observed increase in omadacycline exposure was 88% of the predicted level (Table 3). Analyses using a power model showed that the slope between 300-mg and 600-mg dosing on day 5 was 0.824 (90% confidence interval [CI], 0.607 to 1.041) for AUC_0–24_, falling outside the limits for dose proportionality (Table 4). Analysis of *C*_max_ values, using analysis of variance (ANOVA) and a power model, similarly demonstrated that omadacycline concentrations were dose linear, but less than dose proportional, in this study (Tables 3 and 4).

**TABLE 3:** Statistical analysis of dose-normalized omadacycline pharmacokinetic parameters on days 1 and 5 of dosing^*a*^

Day and parameter	Treatment (mg)	*n*	Geometric LS means	Treatment comparison	Ratio of geometric LS means (%)	90% CI of ratio
Day 1						
AUC_0–24_/dose, ng · h/ml/mg	300	25	21.32			
	450	24	18.64	450/300	87.44	77.41–98.77
	600	24	16.18	600/450	86.79	76.71–98.20
				600/300	75.89	67.20–85.71
*C*_max_/dose, ng/ml/mg	300	25	2.09			
	450	24	1.81	450/300	86.71	76.17–98.71
	600	24	1.54	600/450	85.26	74.76–97.23
				600/300	73.92	64.95–84.14
Day 5						
AUC_0–24_/dose, ng · h/ml/mg	300	23	30.09			
	450	24	28.83	450/300	95.82	90.39–101.59
	600	23	26.46	600/450	91.78	86.58–97.30
				600/300	87.95	82.96–93.25
*C*_max_/dose, ng/ml/mg	300	23	2.62			
	450	24	2.32	450/300	88.58	83.19–94.32
	600	23	2.11	600/450	90.72	85.20–96.60
				600/300	80.36	75.47–85.58

a Results are for PK population and were determined by ANOVA; see Materials and Methods for details. One subject during 300-mg omadacycline dosing and 1 subject during 600-mg omadacycline dosing vomited before reaching PK steady state on day 5. These subjects met criteria for exclusion from PK analyses and are not included in the day 5 summary. CI, confidence interval; LS, least squares.

**TABLE 4:** Dose linearity assessment of omadacycline pharmacokinetic parameters on day 5 of dosing^*a*^

Parameter	*n*	Estimated intercept (*a*)	Estimated slope (*b*)	Standard error of slope	90% CI of slope
AUC_0–24_, ng · h/ml	70	4.406	0.824	0.130	0.607–1.041
*C*_max_/dose, ng/ml	70	2.740	0.687	0.129	0.472–0.902

a Results for PK population. One subject during 300-mg omadacycline dosing and 1 subject during 600-mg omadacycline dosing vomited before reaching PK steady state on day 5. These subjects met criteria for exclusion from PK analyses and are not included in the day 5 summary.

Statistical analyses also revealed accumulation of omadacycline in plasma following once-daily dosing for 5 consecutive days. Depending on dose, accumulation ratios between day 5 and day 1 ranged from 1.40 to 1.62 for AUC_0–24_ and from 1.24 to 1.35 for *C*_max_ (data not shown). These findings are consistent with the long *t*_1/2_ of omadacycline in plasma.

### Safety and tolerability.

Overall, 12 of the 33 subjects in the safety population reported a total of 36 TEAEs during the study (Table 5). TEAEs were reported by 38.5% of subjects who received omadacycline and by 28.6% of subjects who received placebo. The highest percentage of TEAEs was classified as gastrointestinal (GI) disorders. The most frequently reported TEAE was nausea, which was reported in ≤7.7% of subjects during 300-mg and 450-mg omadacycline dosing and by 16.7% of subjects during 600-mg dosing.

**TABLE 5:** Summary of treatment-emergent adverse events^*a*^

Parameter	Value(s) for:				
	Omadacycline dose			Omadacycline overall (*n* = 26)	Placebo overall (*n* = 7)
	300 mg (*n* = 26)	450 mg (*n* = 24)	600 mg (*n* = 24)		
No. (%) with any TEAE	5 (19.2)	3 (12.5)	6 (25.0)	10 (38.5)	2 (28.6)
No. (%) with treatment-related TEAE	4 (15.4)	2 (8.3)	6 (25.0)	9 (34.6)	1 (14.3)
Most frequent TEAEs (seen in >1 study subject), *n* (%)					
Nausea	2 (7.7)	1 (4.2)	4 (16.7)	6 (23.1)	0
Vomiting	2 (7.7)	0	1 (4.2)	3 (11.5)	0
Diarrhea	0	0	2 (8.3)	2 (7.7)	0
Dizziness	2 (7.7)	0	1 (4.2)	3 (11.5)	0
ALT increased	0	1 (4.2)	1 (4.2)	2 (7.7)	0
TEAEs leading to early discontinuation of study drug, *n* (%)					
All	1 (3.8)	1 (4.2)	1 (4.2)	3 (11.5)	1 (14.3)
Nausea	1 (3.8)	0	0	1 (3.8)	0
Vomiting	1 (3.8)	0	0	1 (3.8)	0
ALT increased	0	1 (4.2)	0	1 (3.8)	0
Lipase increased	0	0	1 (4.2)	1 (3.8)	0
Syncope	0	0	0	0	1^*b*^ (14.3)

a Results for safety population.

b Vasovagal syncope following a blood draw.

There were no serious TEAEs reported during the study. Four subjects experienced TEAEs leading to study discontinuation, including 1 subject during each of the 3 omadacycline dose levels and 1 subject in the placebo group.

There were no clinically significant findings in vital sign measurements, physical examination, electrocardiogram (ECG) results, hematology, or urinalysis parameters. Serum chemistry analyses showed that between baseline and day 5 of each dosing period, the median change in alanine aminotransferase (ALT) concentration was −2.0, 5.0, and 19.5 IU/liter in subjects dosed with 300 mg, 450 mg, and 600 mg omadacycline, respectively. The corresponding changes in placebo groups ranged from −5.0 to −1.0 IU/liter. No substantial changes in median aspartate aminotransferase (AST), bilirubin, or other serum chemistry parameters were noted. The highest individual ALT value was 150 IU/liter (2.7-fold above the upper limit of normal [ULN]), which occurred in a subject who first received 450 mg omadacycline in period 1, 300 mg in period 2, and then was discontinued due to the liver enzyme changes; this subject's bilirubin values remained within the normal range at all time points assessed.

## DISCUSSION

Although a number of phase 1 to 3 studies support once-daily 300-mg oral omadacycline as a safe and effective therapeutic dosing regimen, it is possible that greater drug exposure from higher oral doses could be beneficial in some clinical situations. However, multidose regimens of oral omadacycline using doses higher than 300 mg have not been previously investigated. The goal of this study was to understand the pharmacokinetics and safety of oral omadacycline at doses higher than 300 mg. A 3-period crossover study was used to investigate the pharmacokinetics and safety and tolerability of oral omadacycline when administered once daily at dose levels of 300, 450, or 600 mg for 5 consecutive days. The highest dose level was selected based on a previous study in which single oral doses of up to 600 mg were administered to humans and showed an acceptable safety profile, although there was some increased incidence of GI adverse events at doses over 400 mg (unpublished data). The crossover study design was intended to control for intrasubject variability, and based on previous indications of an ∼17-h *t*_1/2_ for oral omadacycline (7), a 5-day washout interval between dosing periods was expected to be sufficient to prevent observation of any carryover effects of the study drug between treatment periods. It is recommended that oral omadacycline be administered in a fasted state due to reduced oral bioavailability when omadacycline is administered within 2 to 4 h of food (10). As such, this study was conducted in fasted subjects.

Twenty-six healthy adult subjects received omadacycline in this study, and 7 subjects received matching placebo. Analysis of plasma samples collected from omadacycline-treated subjects at various time points on days 1 and 5 of each 5-day dosing period showed that mean concentrations of omadacycline peaked at 2.5 h and remained measurable up to 24 h (the last tested time point) at all omadacycline dosing levels (300, 450, and 600 mg). Two subjects experienced vomiting before reaching PK steady state on day 5 and were excluded from the day 5 analysis. On day 5, mean steady-state exposure (AUC_0–24_) in subjects dosed with 300 mg omadacycline was 9,267 ng · h/ml, which is consistent with results of previous studies with 300-mg oral dosing (1, 7). Both AUC_0–24_ and *C*_max_ increased with increasing dose and were nearly, but somewhat less than, dose proportional (ranging from 76% to 96% of expected values across all dose comparisons for AUC_0–24_ and from 74% to 91% of expected values across all dose comparisons for *C*_max_). This was the case on both day 1 and day 5 of dosing. Due to its relatively long *t*_1/2_ (mean of ∼13 h on day 1, ∼16 h on day 5), omadacycline accumulated in plasma over the course of 5 consecutive days of dosing. Thus, at all tested dose levels, systemic exposure on day 5 was ∼50% higher than that on day 1. This degree of accumulation is also consistent with that observed following multiple once-daily dosing of intravenous (i.v.) or oral formulations of omadacycline in early pharmacology studies (1).

As would be expected for most pharmacologic agents, increasing omadacycline dosing beyond a certain point appears to have adverse effects in terms of safety and tolerability. Multiple doses of 300, 450, and 600 mg all were generally well tolerated in this study (all TEAEs were either mild or moderate in severity); however, there were some differences between doses. The frequency of treatment-related TEAEs did not increase with an increase in omadacycline dose from 300 to 450 mg (15.4% versus 8.3%), but such events were more frequent with 600-mg dosing (25.0%). Within the most frequent class of TEAEs, GI disorders, the only 2 reports of diarrhea in this study occurred with 600-mg dosing, and nausea occurred with an incidence at least 9% higher at the 600-mg dose level than at the lower doses. In addition, serum chemistry analyses showed a small but notable dose-dependent increase in median ALT concentrations. Although no individual ALT values exceeded 3-fold the ULN, the higher median at 600 mg suggests an increased chance of more significantly elevated serum transaminase levels with this dose. Based on these observations, for situations in which an oral dose above 300 mg may be beneficial, 450 mg is the oral dose most likely to provide higher omadacycline exposure with favorable safety and tolerability.

In terms of optimizing systemic exposure, this study showed that omadacycline plasma concentrations on day 1 of 450-mg dosing were similar to those on day 5 of 300-mg dosing (mean AUC_0–24_ of 8,976.5 and 9,267.2 ng · h/ml, respectively). For indications in which the therapeutic dosing regimen incorporates 300-mg daily oral dosing, these data support a strategy of using an initial oral loading dose of 450 mg once daily for 1 to 2 days, followed by 300-mg once-daily oral dosing. Such a strategy could eliminate the need for an i.v. phase of treatment and was evaluated in a recently completed phase 3 trial of oral-only omadacycline treatment in patients with ABSSSI (ClinicalTrials registration no. NCT02877927).

In summary, these data indicate that systemic drug exposure to omadacycline increases with increasing once-daily oral dosing from 300 mg to 450 mg or 600 mg, but the exposure is not dose proportional. There were no substantial differences in the safety or tolerability of 300- and 450-mg doses, but increasing the dose to 600 mg appears to have less favorable safety and tolerability. Overall, these data provide information about the pharmacokinetics and safety of oral omadacycline at doses greater than 300 mg and support the potential clinical utility of a 450-mg oral dose of omadacycline, either as part of a loading dose strategy or for indications where systemic exposure higher than that achieved with a 300-mg oral dose is necessary.

## MATERIALS AND METHODS

This study was conducted in accordance with International Council for Harmonisation Harmonised Tripartite Guideline E6(R1): Good Clinical Practice (11). All aspects of the study complied with all national, state, and local laws and regulations. The study protocol was reviewed and approved by the Institutional Review Board at the study center. Each participating subject provided written informed consent prior to enrollment.

### Study design.

This was a phase 1, randomized, double-blind, 3-period, crossover study in healthy adult subjects aimed at evaluating the pharmacokinetics (PK) (primary objective) and safety and tolerability (secondary objective) of multiple once-daily oral doses of omadacycline (at dose levels of 300, 450, and 600 mg). Placebo-treated subjects were included in the study to minimize potential bias in assessing tolerability. The study was performed at a single center, *viz*., PPD Phase I Clinic in Austin, Texas. The study consisted of a screening period (day −21 through day −2), three baseline periods (day −1 of each period), 3 treatment periods (day 1 through day 6 of each period), and a study completion visit (within 6 to 10 days after the last dose of study drug in period 3). Washout periods of at least 5 days were included between the last dose in one period and the first dose in the next period. Subjects were confined to the study site from day −1 of period 1 until discharge on day 6 of period 3, after the 24-h blood sampling and safety assessments were completed.

Subjects meeting the criteria for enrollment in the study (see below) were randomly assigned to 1 of 3 treatment sequences using a Latin square design. Sequences designated which omadacycline dose was administered in each period: 300/600/450 mg, 450/300/600 mg, or 600/450/300 mg in periods 1, 2, and 3, respectively. Subjects assigned to omadacycline received omadacycline during all 3 periods and at all tested dose levels. Subjects assigned to placebo received placebo during all 3 periods. Investigators and subjects were blind to whether the subject received omadacycline or placebo. The study was planned for 30 subjects (24 omadacycline, 6 placebo) divided equally among the 3 treatment sequences (8 omadacycline and 2 placebo treatments per sequence).

Subjects received the appropriate dose of omadacycline or placebo once daily on day 1 through day 5 of each period. Omadacycline 150-mg tablets were used in the study; thus, doses consisted of 2, 3, or 4 tablets (for 300-, 450-, and 600-mg doses, respectively). An equal number of placebo tablets was administered to corresponding placebo groups. All doses of study drug were administered in the morning with no food or drink except for water at least 6 h before dosing. Subjects had no food or drink except water for at least 2 h after dosing and no dairy products, antacids, or multivitamins for 4 h after dosing.

### Subject selection.

Healthy, nonsmoking, male and female subjects were eligible for participation in the study if they were between 18 and 55 years of age (inclusive), weighed ≥50 kg, had a body mass index between 18 and 30 kg/m^2^ (inclusive), met all eligibility criteria during screening (performed within 21 days before dosing in period 1) and at baseline (day −1) for period 1, and provided written informed consent. Health status was determined by past medical history, clinical laboratory tests, vital signs (oral body temperature, systolic blood pressure, diastolic blood pressure, and heart rate), 12-lead electrocardiogram (ECG), and physical examination at screening. Eligibility criteria included ability to swallow up to 4 tablets in succession. Subjects were excluded from participation in the study for prior treatment with omadacycline or recent use of other investigational drugs; ECG abnormalities; inability to tolerate oral medications; pregnancy or breastfeeding; use of tobacco products, prescription drugs, herbal supplements, or over-the-counter medications or intake of xanthine (e.g., caffeine)-containing food or beverages within a specified time frame before study initiation; blood loss/donation; low hemoglobin levels; high creatinine or blood urea nitrogen levels; urinary obstruction/difficulty voiding; positive alcohol or drug test; hypersensitivity or allergy to any tetracycline; signs of liver disease or liver injury; significant illness within 2 weeks of study initiation; any planned medical intervention that might interfere with the study; or a history of diseases or medical conditions as specified in the study protocol.

### Study assessments: plasma pharmacokinetics.

Serial blood samples for PK analysis of omadacycline were collected prior to dosing (predose) and at the following time points after dosing on day 1 and day 5 of each period: 0.5, 1, 1.5, 2, 2.5, 3, 4, 6, 8, 12, 16, and 24 h. In each period, the 24-h blood sample for day 1 was collected before administration of the day 2 dose. The PK analysis population consisted of subjects who received omadacycline and had at least 1 evaluable PK parameter. Subjects were excluded from PK analysis on a given day if they missed doses, had diarrhea, or had vomiting within twice the median *T*_max_ of omadacycline.

Noncompartmental PK parameters were determined from plasma omadacycline concentration and actual time data using Phoenix WinNonlin, version 6.2.1 (Certara, Princeton, NJ, USA), including area under the plasma concentration-versus-time curve (AUC) from 0 to 24 h after dosing (AUC_0–24_), AUC from time zero to the last quantifiable concentration (AUC_last_), maximum observed plasma concentration (*C*_max_), time to reach maximum observed plasma concentration (*T*_max_), terminal elimination half-life (*t*_1/2_), terminal phase rate constant (λ_z_), and the accumulation factor of AUC_0–24_ and *C*_max_.

### Study assessments: safety and tolerability.

Safety assessments included monitoring of adverse events (AEs), clinical laboratory test results, vital sign measurements, 12-lead ECG results, and physical examination findings. All randomly assigned subjects who received at least 1 dose of any study drug (omadacycline or placebo) were included in the safety analysis population. Adverse events were coded by preferred term and system organ class using MedDRA, version 17.1.

### Statistical analysis.

Plasma concentration data were summarized by day and time point for each treatment using descriptive statistics (number of subjects, means, standard deviations, coefficient of variation, median, minimum [min], and maximum [max]). All further statistical analyses were performed using SAS software (SAS Institute, Cary, NC, USA), version 9.2. A linear, mixed-effect, ANOVA model (SAS PROC MIXED) with treatment (300, 450, and 600 mg), sequence, and treatment period as fixed effects and subject nested within sequence as a random effect were fitted to the natural log-transformed dose-normalized PK parameters AUC_0–24_/dose, AUC_last_/dose, and *C*_max_/dose after dosing on day 1 and day 5 of each period for use in estimation of effects and construction of confidence intervals (CIs). Point estimates and 90% CIs for differences on the log scale were exponentiated to obtain estimates for the ratios of geometric means and respective 90% CIs on the original scale. No adjustment was made for multiplicity.

Dose linearity across all 3 dose levels was assessed by fitting omadacycline *C*_max_, AUC_last_, and AUC_0–24_ after both the day 1 and day 5 doses to a power model (12): ln(PK) = *a* + *b* × ln(dose) + error, where PK was the PK parameter, *a* was the intercept, and *b* was the slope. The estimates of slope *b* were reported along with the corresponding 2-sided 90% CIs. If the 90% CIs of the slope, defined by the power model, were contained within the dose proportionality bounds of 0.80 to 1.25 (0.68 to 1.32, when adjusted for dose), dose proportionality over the 300- to 600-mg dosing range was concluded.

For statistical analysis of accumulation of omadacycline, a linear mixed-effect model with day as a fixed effect and subject as random effect was fitted to the natural log-transformed *C*_max_ and AUC_0–24_ to construct 90% CIs for day 5 compared with day 1 (at each dose level separately).
